# Forwarding in Energy-Constrained Wireless Information Centric Networks

**DOI:** 10.3390/s22041438

**Published:** 2022-02-13

**Authors:** Daniel Marques, Carlos Senna, Miguel Luís

**Affiliations:** 1 Instituto de Telecomunicações, 3810-193 Aveiro, Portugal; danielfmarques@av.it.pt (D.M.); cr.senna@av.it.pt (C.S.); 2 ISEL—Instituto Superior de Engenharia de Lisboa, Instituto Politécnico de Lisboa, 1959-007 Lisboa, Portugal

**Keywords:** energy consumption management, content forwarding, information-centric networks, internet of things, smart city

## Abstract

Information Centric Networks (ICNs) have been considered one of the most promising candidates to overcome the disadvantages of host-centric architectures when applied to IoT networks, having the potential to address the challenges of a smart city. One of the foundations of a smart city is its sensory capacity, which is obtained through devices associated with the IoT concept. The more sensors spread out, the greater the ability to sense the city. However, such a scale demands high energy requirements and an effective improvement in the energy management is unavoidable. To improve the energy management, we are proposing an efficient forwarding scheme in energy-constrained wireless ICNs. To achieve this goal, we consider the type of devices, their internal energy and the network context, among other parameters. The proposed forwarding strategy extends and adapts concepts of ICNs, by means of packet domain analysis, neighbourhood evaluation and node sleeping and waking strategies. The proposed solution takes advantage of the neighbourhood to be aware of the moments to listen and forward packets in order to consistently address mobility, improving the quality of content delivery. The evaluation is performed by simulation with real datasets of urban mobility, one from the lagoon of “Ria de Aveiro” and the other from a vehicular network in the city of Porto. The results show that the proposed forwarding scheme resulted in significant improvements in network content availability, in the overall energy saving and, consequently, in the network lifetime.

## 1. Introduction

In recent years we have seen a radical change in the way devices connect to the Internet. The best example is the IoT, a recent communication paradigm in which objects of everyday life will be able to, among others, communicate with one another, becoming an integral part of the Internet. In this context, wireless networking is expected to sustain the direct interaction between personal users’ devices and also provide connectivity on a large scale for resource-constrained devices. However, conventional networking protocols, such as the traditional Transmission Control Protocol/Internet Protocol (TCP/IP) host-centric network, fail in large scale mobile wireless distributed environments, such as IoT scenarios, due to node mobility, dynamic topologies and intermittent connectivity, to name a few [1].

Information Centric Networks (ICNs) [2] have been considered one of the most promising candidates to overcome the drawbacks of host-centric architectures when applied to IoT networks. Conceptually, in ICNs, each piece of data has a unique, persistent and location-independent name that is directly used by the applications for content search and retrieval. Therefore, ICNs enable the deployment of in-network caching and content replication, thus facilitating the efficient and timely delivery of information. These features increase the challenge in building efficient energy management solutions for ICNs, as traditional solutions for IP networks are of little value in ICN-based environments. In this way, some questions arise: In mobile energy-constrained and multi-technology environments, which interface should be preferred for content resolution and content forwarding? Should we replicate all the Interest packets back from the same interface or should some contention be adopted to avoid unnecessary transmissions? Should energy-constrained devices adopt a conservative approach?

To answer these questions, we are proposing a forwarding strategy for energy-constrained wireless ICNs. Our solution considers the type of device, network context and ICN caching, among other parameters, to implement new content resolution for mobile wireless networks. The proposed forwarding strategy extends and adapts concepts of ICNs, by means of packet domain analysis, neighbourhood evaluation and node sleeping and waking strategies to increase the energy saving and reduce the use of resources in unnecessary situations. This strategy is particularly important in this scenario because IoT nodes may have small and limited batteries. Furthermore, the proposed solution takes advantage of each node’s neighbourhood to be aware of the optimal moments to listen and forward packets in order to consistently address mobility, improving the quality of content delivery.

To bring our evaluations one step closer to a real scenario, we use two real traces of urban mobility, one with boat mobility, gathered from the Aveiro lagoon “Ria de Aveiro”, in the city of Aveiro, Portugal, and the other from a vehicular network running in the city of Porto, Portugal, gathered from public buses from the Porto Collective Transport Society (STCP). STCP’s buses form a sensing and data distribution platform composed of 600+ public transport vehicles equipped with On-Board Units (OBUs) for V2X (Vehicle to Everything) communications and in-vehicle internet connectivity for passengers. The mobility traces of STCP buses were collected through the Porto Living lab IoT platform [3] and the mobility of boats was collected by communication infrastructure of the Aveiro STEAM city project (https://uia-initiative.eu/en/uia-cities/aveiro (accessed on 28 January 2022)).

The results show that the energy-aware proposed forwarding scheme resulted in significant improvements in network content availability, in the overall energy saving of the nodes and, consequently, in the increase in the network lifetime. Moreover, it was possible to save energy previously spent in listening stages and forwarding packets in situations of no connectivity, resulting in an increase of 25 min of network lifetime in the Aveiro scenario and almost 5 h in the Porto scenario. The main contributions of our work are:

- Design of an efficient forwarding strategy for energy-constrained wireless ICNs that takes into account inputs from the network structures, as well as context, neighbouring environment and energy level in the decision process;
- Modification of the Named Data Networking (NDN) framework and the network simulator 3 (ns-3) energy framework, along with the redesign of the logging module, to include the proposed forwarding strategy;
- Assessment of the overall network performance and power consumption in IoT scenarios considering two mobility patterns.

The remainder of this paper is organised as follows: Section 2 discusses the related work; Section 3 shows the basic characteristics of the ICN paradigm; Section 4 details the proposed efficient forwarding in relation with energy management; Section 5 describes the scenarios and metrics and discusses the results obtained by simulation using real mobility traces; and, finally, Section 6 enumerates the conclusions and points out the future work.

## 2. Related Work

In the last years, several works have investigated a way to minimise the large broadcast storm problem and reverse path partitioning in mobile networks [4,5,6]. However, there are few works that take into consideration the energy-constrained wireless ICNs, more specifically, the energy use and management of each node in the routing decision process.

In the work of [7], a forwarding strategy was proposed to select a node from its neighbourhood as a cluster head responsible for forwarding the Interest packets, based on the information regarding the satisfied Interest rate between the neighbouring nodes. This required the addition of two new data structures, one to store these neighbour values and another to store its own values. In this case, the security barrier is broken by using the extraneous information of neighbouring nodes, a list of nodes that have a higher satisfied Interest rate. In the strategies of [8,9], the Global Positioning System (GPS) positions are used by a geolocation mechanism that routes the Interest packets to the Producers. By using the Producer position, the content-centric paradigm ceases to exist, becoming host-centric. Moreover, in highly mobile environments, this Producer position is compromised, as there may be mobile Producers, not maintaining the same position over time. The works of [7,10] elect a node from their neighbourhood to forward their Interest packets according to their data delivery success rate. This requires adding new fields and structures for these values to be stored.

Although these solutions improve content accessibility, transmission reliability and robustness and minimise the broadcast storm problem, they still change the basics of the ICN architecture, bringing more complexity and overhead to the network. Therefore, it is essential to develop new and more efficient forwarding strategies that guarantee higher network performance while maintaining the basic ICN architecture.

On the other hand, the work in [11] proposes two mechanisms for packets transmission aiming to minimise the broadcast storm problem and the energy consumption. Two forwarding modes are combined and switched based on the lookup performed by the Forwarding Information Base (FIB) and efficient mechanisms are implemented to control flooding in order to save energy. However, this proposal does not consider mobility and very specific and static scenarios are evaluated. Similarly, [12] uses greedy packet forwarding, using Hello packets to send its neighbourhood information (ID, location and remaining power). In this proposal, the security barrier is broken through the sharing of confidential information from each node. The works [13,14] implement a sleep scheduler that is based on monitoring information from the environment and the operating state of the nodes, choosing one of two states (semi-sleep or sleep) at random times. However, this work is designed for Wireless Sensor Networks and does not apply to ICNs. The work in [15] proposes a multihop cooperative caching scheme for green wireless sensor networks over receiver-triggered ICNs. Using regional caches, the proposal saves energy while reducing the delay in fetching data. In [16], a Power Saving Mode is proposed to manage the time spent in idle listening state, consisting of a state machine (transmit, receive, idle, sleep) that is based on the Pending Interest Table (PIT) state and of a beacon that is sent by the Access Points containing the Traffic Indication Map (a list of nodes that have to wake up to be ready to receive data). In this proposal, the network depends on the beacon that is sent by the APs to be able to decide if the node should wake up to receive or transmit the content. The solution cannot be applied in fully mobile environments, where there is no interaction with a static network element and where mobile nodes may experience a long period of disconnection from the network. Furthermore, it is necessary for such beacons to circulate through the network and constantly update themselves, which increases the network overhead.

The works of [17,18] use a Software-Defined Network (SDN) that supports the ICN approach by having the knowledge of the network topology, communicating any information to the nodes and update routing information. The SDN also helps in efficient neighbourhood discovery and in the communication of the new nodes’ positions. However, this architecture brings more complexity to the network system using external resources. Moreover, the communication of the new positions of certain nodes brings security issues and ends up with spatial and temporal non-reference of the nodes.

An important restriction found in the aforementioned solutions is the addition of new fields in the packets (Interest and Data), addition of new tables and structures to the native ICN architecture. These additional fields, which are added to the packets that will be transmitted through the network, usually refer to parameters or properties of the nodes, such as their position and remaining energy, which should be private information of each node. Many of the works conducted that achieve improvements in network energy saving do not take into account the mobility of the nodes or overcome the security barrier and exploit the positions of the Producers to route the Interest packets in the right direction, which exposes sensitive node information that should be private. Furthermore, they lack practical evaluation using real-world scenarios with resource-constrained mobile elements.

Our proposal aims to solve the various problems mentioned before. It does not compromise security with the sharing of information among the nodes, it is intended for application in highly mobile ICN environments, manages the nodes’ internal energy in the packet forwarding decision process and maintains the basics of the ICN architecture without adding new fields to packets, creating new tables or changing structures. Finally, we evaluated our mechanisms and strategies using real data from boat and bus movements gathered from the cities of Aveiro and Porto, Portugal.

## 3. ICN Basics

**Figure 1:**
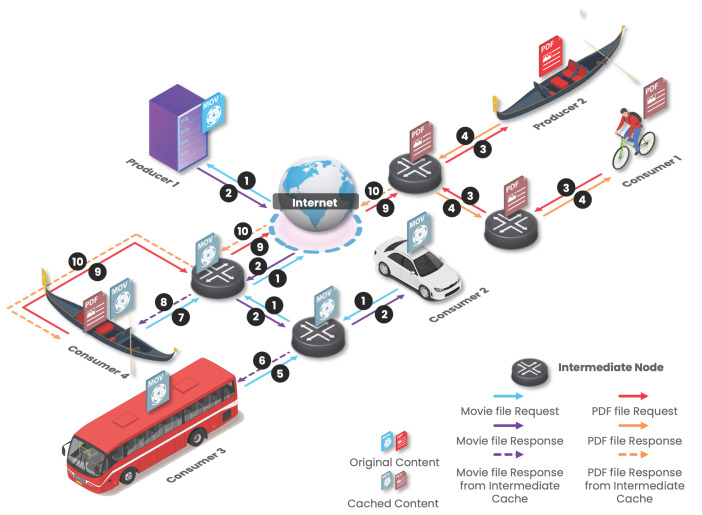
ICN operation mode. Consumers request specific content which is initially stored in the Producers. Then, as this content passes through the intermediary nodes, it is retained in the cache for faster response to future requests.

In an ICN architecture, the Consumer makes a request for content without knowing where its Producer is. The communication between the Consumer and the Producer follows a request–response exchange model with native support of multicast, which can be especially useful both in a Vehicular ad hoc Network (VANET) environment and Internet of Things (IoT) [19]. During this process, as each piece of data has a unique name, persistent and independent of their location, the system takes care of mapping the requested data to their originating location, which facilitates applications in the process of searching and collecting future data with the same name. When the request arrives at the Producer’s side, the requested Data follow the reverse path through which the request arrived, following a receiver-oriented approach. ICN has solved the need for in-network caching by replicating content across network nodes to improve content delivery by being faster and more efficient, as can be seen in Figure 1, where the mode of operation of an ICN is explained.

In the example of Figure 1, Consumers request content (be it a PDF document or a movie) initially stored at the respective Producers. The content request is forwarded to the Producers, and when the content comes back by the reverse path of the Interest packet, the content is cached in the intermediate nodes, enabling the content satisfaction of future requests.

ICN does not use source-destination host pair communication. Data are identified by unique names [20]. Naming schemes can be hierarchical, where content names are human readable and can be hierarchical and structured, such as Uniform Resource Locator (URL) [21]; flat, where names the contents use are small, unique and self-certified names (hashes, for example) [22]; attribute-based, where names are expressed through keywords that are extracted from the contents as attributes, such as type, version; and hybrid, where names the contents are using are a combination of all the schemes mentioned before to take advantage of the best parts of each one [23]. An example name for content that circulates a smart city network could be //SmartCityUrbanPlanning/TrafficManagement/Decision/“ContentContext” /:::“ContentAttributes”.

The ICN architecture uses two types of packets in all network communication: the Interest packet and the Data packet [2]. The Interest packet is used to define a request for a particular content and contains two mandatory fields: the Name, which identifies the intended content, and the Nonce, which uniquely identifies the packet circulating over the network. It may also contain Selectors that serve as input parameters to the routing and content resolution mechanisms, such as the indication that this Interest may receive stale Data, the lifetime of the Interest, the hop count limit before the Interest is discarded, among others. The creation of the Data packet is associated with the reception of an Interest packet, so the Name is the same in both packets (it is extracted from the Interest packet). It also has the data itself represented by content and the Signature that is divided into SignatureInfo, which is included in the calculation of the signature and describes itself, and SignatureValue, which is excluded from the signature calculation and represents the signature material.

ICN provides temporary storage of content on router nodes that can meet future requests [24]. Data packets are available closer to the Consumer, reducing the number of packets that travel through the network. This allows the reduction in the bottleneck of messages exchanged, greater availability of content spread over the network, reduction in response latency and, of course, energy savings.

**Figure 2:**
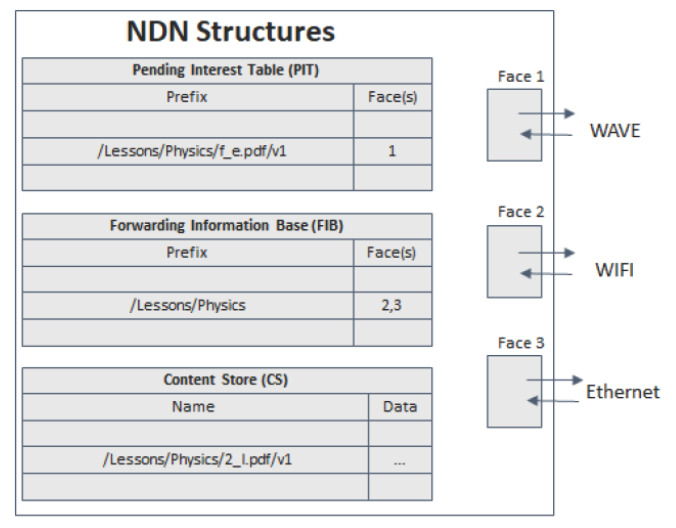
NDN data structures.

One of the most promising architectures based on the ICN concept is the Named Data Network (NDN) [2]. The NDN architecture makes use of hierarchically structured content names, content source reliability (through authentication by the content provider through cryptographic mechanisms such as public keys) and a routing protocol that executes forwarding mechanisms using names instead of IP addresses. Due to the facilities provided by the NDN project [25], especially the NDN simulator (ndnSIM) [26], this architecture was the one we chose for the development of this work. The NDN allows users to make a request for data without knowing a priori which entity produces or possesses it. It also allows user mobility and security issues to be handled more efficiently than the current Internet Protocol (IP). The entities that exchange information in the NDN network can be separated into Producers, Consumers and router/intermediate nodes, forwarding the Interest and Data packets between Consumers and Producers. It is important to say that a node can assume all three roles concurrently. A node belonging to an NDN network maintains three important data structures: the Forwarding Information Base (FIB), the Pending Interest Table (PIT) and the Content Store (CS), illustrated in Figure 2.

The FIB is a table of name prefixes with their output interfaces that allows the forwarding of Interest packets by searching by their name. The PIT is a table that records the Interests that are requested, that are waiting for Data, and the interfaces through which they came. The CS is a cache for incoming Data packets, which can be used for faster responses to future Interests of that same Data. The Faces are the interfaces that receive and forward the packets.

**Figure 3:**
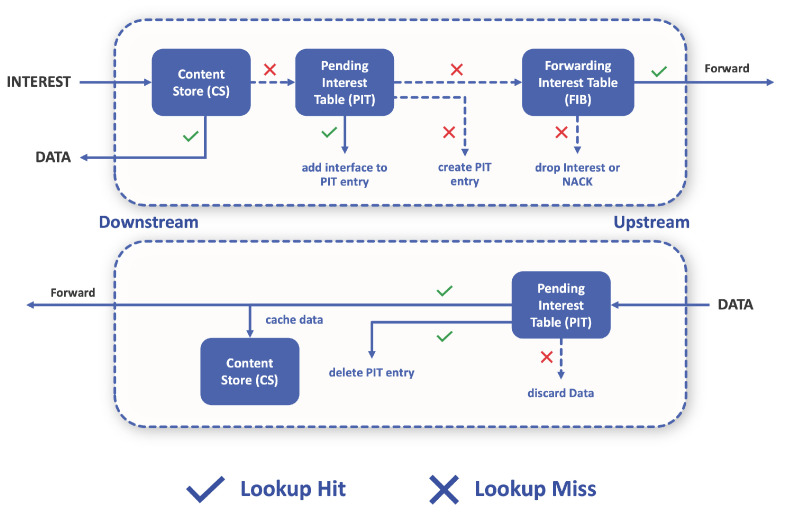
Forwarding process in NDN.

Let us now look on how these structures are used in the forwarding process. As shown in Figure 3, packet forwarding can follow an upstream or downstream direction according to the information present in the data structures and the type of packet concerned. When an Interest arrives at a node (upstream), the CS is checked for the intended Data. If the Data are not in the CS, the Interest is forwarded to the PIT where it is stored along with the interface through which it arrived (if this entry already exists, only the interface is stored and the Interest is discarded), and then uses the FIB to forward this packet if possible (otherwise it is discarded). If an Interest reaches a node that has the Data in the CS, that same node replies with the desired Data along with a signature from the Producer. This Data packet then follows the reverse path of the Interest packet, until it reaches the Consumer that requested that Data. The same procedure is performed with the Negative Acknowledgement (NACK) packets that will notify the Consumer of some congestion, duplication of Interest or in case no route was found.

## 4. Energy-Efficient Wireless NDN Architecture

**Figure 4:**
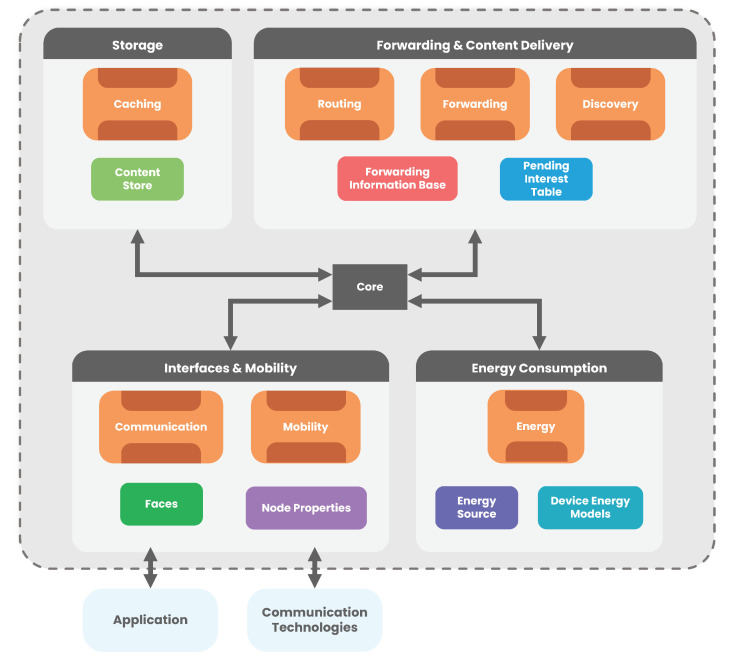
Energy-efficient wireless NDN-based architecture.

The main advantage of using the NDN design in a highly mobile environment is that it increases the availability of content on a network. Excessive power consumption in dense IoT scenarios brings problems to the forwarding process, causing unnecessary packet delays and decreasing network performance. To solve these problems, we propose strategies that aim to minimise excessive energy consumption, optimising the processes without harming the NDN functionalities. Figure 4 illustrates an NDN macro architecture with the main components of the base architecture (Storage, Forwarding and Interfaces and Mobility) [2] encompassing the proposed *Energy Consumption* component that supports the solution for efficient energy management.

The central core module is responsible for the interaction between all the modules, allowing their logical and efficient operation. The Storage represents the Caching process of a node, which aims to store the contents in memory to be able to respond to future requests for the same contents. The Forwarding and Content Delivery is composed of three processes: Routing, Forwarding and Discovery. The Routing is responsible for keeping a register of the available paths to a given content. Forwarding allows the routing of Interest and Data packets in an efficient way through the available paths. Discovery complements Routing by determining the most logical path to forward Interest packets if no other path to the content is available. This is to make an effort to find a path to the content first, rather than discarding the packet outright. The main data structures used by these processes are FIB and PIT.

The module Interfaces and Mobility (I&M) consists of Communication and Mobility processes. The Communication is responsible for the connection and communication between devices and the application layer, and between other external devices. The Mobility is related to all node parameters that may have direct or indirect influence on its mobility. It allows the mobile characterisation of any mobile element, which is related to the Node Properties and that are used in the remaining forwarding and caching processes. Besides, the I&M processes use Faces that symbolise the communication interfaces of the NDN Stack and Node Properties that contain the node’s properties, such as the mobility type, among others. Finally, our Energy Consumption module is composed of the Energy component responsible for measuring the energy consumption and how a node should react to certain energy situations, presenting two essential components: the Energy Source and the Device Energy Models. The Energy Source is responsible for the implementation of the power sources or batteries in a node that can be modelled in a linear or non-linear way. The Device Energy Models are associated with the way energy is consumed by different technologies (radio interfaces, processors, sensors, etc.). An example of the information collected and used by Device Energy Models for Wi-Fi technology is [27]: Sleep = 0.00132 A, Transmit/Tx = 0.167 A, Receive/Rx = 0.310 A, Range = 50 m and Frequency = 2.4–5.0 GHz. Below, we highlight the implementations made for an efficient management of the energy resources of the network nodes.

### 4.1. Interfaces and Mobility Module

In the I&M module, we introduce the mechanism responsible for the discovery of the type and number of nodes in the neighbourhood as well as the mechanism responsible for processing the direction of the node to predict situations where energy management can be used.

#### 4.1.1. Neighbourhood Status

The neighbourhood awareness is important for the decision of packet forwarding and for the sleep strategy. The state of a node’s neighbourhood is based on four essential aspects: the available interfaces, the number and type of neighbours, the cost associated to the interfaces and the mobility metrics. A stationary or moving node can have different communication technologies. As a node is constantly moving, the wireless link conditions are constantly changing, and picking the best one to communicate is very important to the forwarding strategy and energy saving, bringing a great value to network efficiency.

The number and type of neighbours (mobile or static) per interface is an important factor, since it determines how many neighbours it has and what type of mobility they present, aspects that are considered when deciding which interface a packet should be forwarded through or whether it is a good time for the node to go in sleep mode.

The Mobility module is responsible for updating neighbourhood parameters, such as mobility type and Received Signal Strength Indicator (RSSI). However, there are other factors that influence the neighbourhood relationship such as the speed and direction of a node. To collect the aforementioned information needed to implement the neighbourhood awareness module, we present a mechanism whose flowchart is presented in Algorithm 1. The mechanism runs every 1 s for each mobile node and checks, for the NetDevice of the wireless interface (LINK_TYPE_ADD_HOC) (line 6), the RSSI value of a given neighbour (line 14) and the type of node (mobile or static) (line 16). In addition, we can also obtain the position of the neighbour (line 10), which can be obtained from the RSSI values [28,29], for example, or from periodic messages transmitted by the nodes through Cooperative Awareness Messages (CAM) of the Intelligent Transportation System (ITS) [30]. At the end, this information is communicated to the Forwarder module (line 16).

#### 4.1.2. Node Heading and Direction

**Figure 5:**
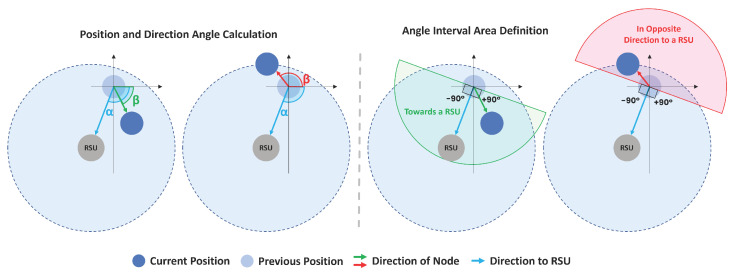
Node heading calculation: two distinct situations.

In order to determine situations where a node can save energy by entering in sleep mode, its direction has been considered. For each static node (Road Side Units—RSU) in the neighbourhood, its position is obtained, and the direction of the static node regarding the previous position of the node is calculated and then converted to an angle in degrees (α). The quadrant is also computed, as illustrated in Figure 5. The next step is the definition of the angles that define the possible directions named as “Towards an RSU” (TRSU) or “Outwards of an RSU” (ORSU). For this, the range of TRSU angles was considered to be all those between the RSU +90${}^{\circ }$ and RSU −90${}^{\circ }$ angle, giving a range of angles with a view of 180${}^{\circ }$. All angles that do not belong to this range are considered as ORSU. Whenever the angle of the node’s direction is within the range TRSU, it means that it is heading towards that RSU. This proposal is very useful to predict the direction of the node and to estimate the time when it may be entering an area without connection/low RSSI.
**Algorithm 1.** Neighbourhood status procedure.  1:**procedure** NEIGHFINDER  2:    number←0                                 ▹ neighbour count  3:    type←[]                                ▹ list of node mobility types  4:    rssis←[]                                      ▹ list of RSSIs  5:    positions←[]                                   ▹ list of positions  6:    **for** each device **do**  7:          **if** device **is** TypeAdHoc **then**  8:              Obtain WifiNetDevice of device  9:              Obtain nodePosition to WifiNetDevice                    ▹ Obtain node position10:              positions(device).insert(nodePosition)                 ▹ insert position on list11:              Obtain phy of WifiNetDevice12:              receiverMobility←phy→GetMobility()13:              Obtain RSSI to receiverMobility                           ▹ Obtain RSSI14:              rssis(device).insert(RSSI)                         ▹ insert RSSI on list15:              nodeType←WifiNetDevice→GetMobilityType()    ▹ Obtain node type (mobile or static)(mobile or static)16:              type(device).insert(nodeType)                         ▹ insert type on list17:              number←number+1                        ▹ neighbour count increment18:        **end if**19:    **end for**20:    Updates number, rssis, and type to Forwarder21:**end procedure**

### 4.2. Energy Consumption Module

Energy management in communication networks is closely linked to the technology used. In Wi-Fi, a node can be in one of three general states: in the *Active Mode* state when a node transmits, receives and is listening for content, in the *Power-Save Mode* state when a node is asleep, with no content being transmitted or received, or in the *Off* state when the node is off [31]. During Active Mode, there are three ways in which power is consumed: in Transmitter/Tx mode, in Receiver/Rx mode or in Listening/Idle mode. On the other hand, during the Power-Save Mode state, the node will be sleeping, without any communication, and it will only consume the energy defined for that state (*Sleep Mode*). Finally, in the *Off Mode* state the node is disconnected, interrupting communication and not having any energy consumption. For each of the states defined above, it is important to define how much energy is consumed by each of them. For Wi-Fi, the following values have been considered: Sleep = 0.00132 A, Transmit/Tx = 0.167 A, Receive/Rx = 0.310 A, Range = 50 m and Frequency = 2.4–5.0 GHz.

The battery level is an important factor to consider in the decision to forward a packet, because in a device with limited portable battery, it is important to guarantee a minimum amount of battery. Whenever there is an update from the power state, the battery level (%) is updated in the Forwarder module of the respective node. In this way, the Forwarder uses this value in the forwarding decision and checks if it exceeds the minimum value.

An important part of any energy saving strategy is how to evaluate situations where the node can or cannot communicate. Situations where the mobile node is isolated or unable to communicate with its neighbours are energy saving opportunities by putting them to sleep. In this scheme, a node is put into sleep mode in two situations. When there are no neighbours, mobile or static, it sleeps for 1 s. After that, it wakes up and checks the channel for neighbourhood. If the same condition remains, it goes back to sleep another second, and so on. The other situation is when a node is within the range of at least one RSU, is heading in the opposite direction to it and exceeds a certain RSSI threshold.

**Figure 6:**
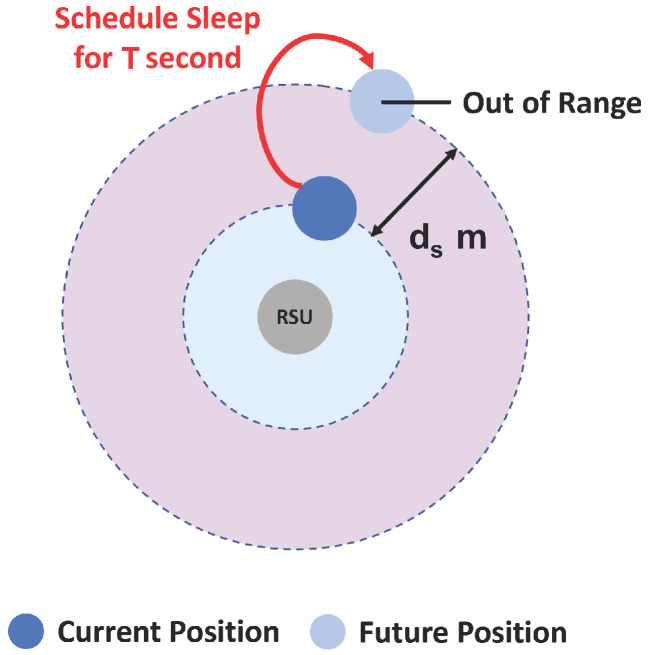
Illustration of the sleeping situation of a mobile node based.

Our proposal defines the RSSI threshold as a function of the distance between the mobile node and the static node [28,29]. The idea is to put the mobile node into sleep when it is expected to leave the wireless range of the static node in the next second. In addition, the sleep time will not be one second, but the amount of time needed by the mobile node to leave the range of the static node. Then, the sleep time *T* is determined by $d_{l}/d_{s}$, where $d_{l}$ is the remaining distance until it reaches the limit of the wireless coverage of the static node and $d_{s}$ is the expected distance travelled in the next second, and is related with the current average speed (Figure 6). Whenever a node finds itself in this condition (within range of at least one static node with low RSSI) but motion is stopped, the node cancels any sleep mode scheduled that it may have and schedules a new one for the new distance to the edge of the communication range. On the other hand, if the node changes direction, it just cancels any sleep mode scheduled. Regarding the transition from sleep mode to “wake up” it can happen in two situations: every second, periodically, and when it has urgent content to transmit.

### 4.3. Forwarding and Content Discovery Module

This module comprises the entire process regarding packet forwarding. Content is discovered through Interest packets, which contain the prefixes within the network domain of the content to be fetched, and this content is retrieved in the form of a Data packet. To make this module suitable for efficient node power management, we added content priority and refined the entire caching and forwarding strategy to make the module energy friendly.

#### 4.3.1. Content Priority

In order to study the degree of importance/urgency of a requested content, they have been divided according to [32], specifying six domains with specific weights:

- **Emergency**. These are the most urgent applications that usually follow a pushed-based approach to be spread across all nodes in the network, being limited in time, such as an accident or dangerous weather condition. It is time constrained, and we give it the value 6.
- **Decision**. These are the applications where requests are important in decision making, being also time limited, such as traffic management. It is time constrained, and we give it the value 5.
- **Information (Stream)**. This includes applications that send and receive data that are limited in time, such as a video stream that should not have latency. It is time constrained, and we give it the value 4.
- **Feedback**. These are the applications that work as data notification, not being limited in time, as is the case of casual meteorological values. It is hybrid, and we give it the value 3.
- **Interaction**. These are the applications connected to the communication between nodes for the exchange of states. It has no time restriction, and we assign the value 2.
- **Information (Data)**. These are the applications that send and receive data which are not limited in time, for instance, the exchange of a file. It has no time restriction, and we assign the value 1.

The weight assigned to each domain distinguishes the degree of importance of a given content base. These values are used in the forwarding decision, along with the different neighbourhood scenarios and the internal energy state of a node. In the content forward decision, values equal to or greater than 4 are considered as priority.

#### 4.3.2. Energy-Efficient Forwarding

**Figure 7:**
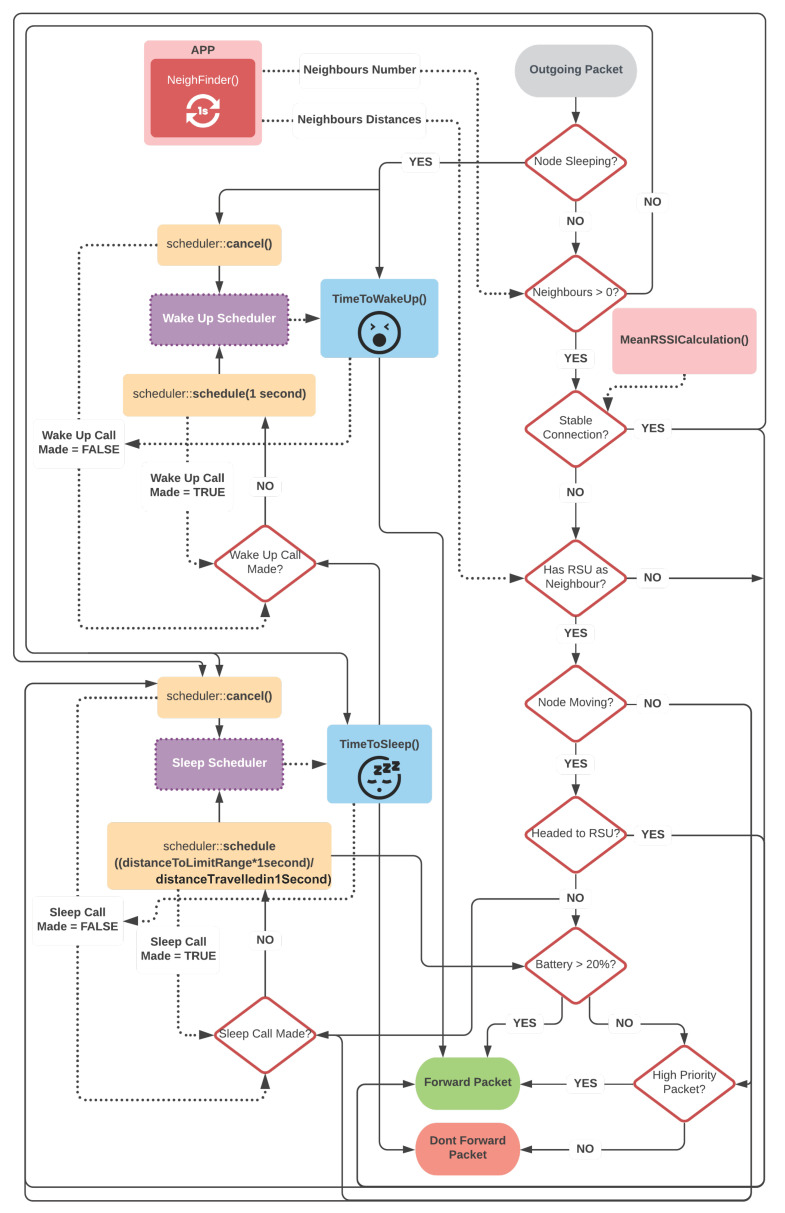
Packet forwarding decision process.

All the aforementioned factors and metrics provide a set of information about the conditions around a node that serves as a heuristic to decide about the forwarding of a given packet. This process is driven by the Forwarding Strategy, through the assessment of the context, energy, neighbourhood, node properties and data structures. The workflow for this decision has been implemented in the same way for both Interest and Data packets and can be seen, in a general and hierarchical way, in Figure 7.

Every time a node has a packet to transmit, it starts by checking whether it is in *Sleep Mode*. If so, it cancels all schedulers that may have been created to wake up and wakes up. Otherwise, it moves to the next phase responsible for updating the neighbouring information. In the case of not having any neighbour, it enters in sleep mode and does not transmit the content. In the case of having a neighbour, and the connection is considered to be stable, the node checks the packet priority and it is transmitted only if the packet weight is higher than 4. If the connection is considered to be unstable, it checks if it is facing a mobile-only neighbourhood or with a single static node. If there are only mobile neighbours, the node forwards everything.

On the other hand, if the node has at least one RSU as a neighbour, the position of the RSU and its direction of movement regarding the mobile node is computer. If the node is stopped (direction 0), it cancels all schedulers that may have been created to sleep and sets a new scheduler to fall asleep after a time *T*, calculated as explained before. In addition, the packet weight is checked and if it is considered priority, the packet is forwarded.

However, if the node is moving, it is checked if the direction of the node is in the direction to the RSU or in the opposite direction. If it is in the direction of the RSU, the node cancels all schedulers that may have been created to sleep and sends the packet. If the node is going in the opposite direction, it checks if any scheduler to fall asleep was made. If not, it sets a scheduler to fall asleep after a time *T* and it checks the remaining battery level. If it is greater than or equal to the predefined threshold of 20%, the packet is forwarded. Otherwise, the packet weight is checked and the packet is only forwarded if it is considered to be priority.

Finally, our solution mainly focuses on predicting transition times between static nodes (RSUs) and dead zones to put some nodes to sleep, thus reducing unnecessary packet forwarding, and listening times, resulting in energy savings. Not only that, but we also use the internal power state of each node to decide in which situations it is most feasible and efficient to send certain packets. This makes each node autonomous and the entire decision making on energy consumption happens in a decentralised way. It is up to the mobile node to decide what to do with the information at its disposal, without the need for any centralised consultation or consultation supported by the static part of the network. Moreover, it is important to highlight that, despite the breadth of innovations added to the NDN’s base architecture, its paradigm has not been changed, i.e., the control structures (PIT, FIB and CS) have not been changed or new types of packets have been created.

## 5. Performance Evaluation

To bring our evaluations one step closer to a real scenario, we use two real traces of urban mobility, one with boat mobility gathered from a lagoon of the city of Aveiro and the other gathered from the vehicular network in operation in the city of Porto, both of them cities of Portugal. For both scenarios, the Data providers/producers are the static nodes (RSUs) and the Data consumers are the mobile nodes (OBUs). Each OBU has only one 802.11n (Wi-Fi) interface, while each RSU has two communication interfaces, an 802.11n (Wi-Fi) interface and a point-to-point connection (Ethernet) with the backend Routers. All IEEE 802.11n interfaces have been configured to have a range of approximately 50 metres, considering that they are in an urban environment, and use the same transmission rate for each packet sent, following a constant data transmission rate of 54 Mbps, while the point-to-point communication interfaces have a data transmission rate of 1 Gbps and a delay of 1 ms. All interfaces of all nodes have a cost of 1 that remains fixed until the end of the simulation.

The Aveiro scenario is composed by the mobility of nine tourist boats (named moliceiros, and simply denoted as OBUs) through the lagoon of the “Ria de Aveiro”. On each route, the boats can head off in different directions, which means that the contact time between them is short and intermittent. These OBUs make contact with four RSUs installed along the boats route and two backend routers to create the connection between all RSUs. These mobility data were collected from 10:35 a.m. to 17:56 p.m. on 23 February 2018. The boats may request Urgent contents, such as information about accidents, adverse weather conditions, among others, or Non Urgent contents, such as Points of Interest (POI) information, traffic, among others.

The Porto scenario uses datasets collected by a vehicular network placed in the city [3]. The dataset with mobility traces is composed by 80 mobile nodes (buses—OBUs) that have circulated during four hours, from 9:00 a.m. to 13:00 p.m., on 23 January 2018. Besides the 80 mobile nodes, this scenario is also composed of a set of 26 RSUs spread throughout the city and 4 backend routers that connect the RSUs. The parameters of both scenarios are represented in Table 1.

The platform used for the evaluation of the proposed forwarding strategy was the ndnSIM [26] software, an Open-Source Simulator Platform extended from the well-established NS-3 research-oriented network simulator. To simulate the energy consumed by each of the PHY states, we defined 90.000 Joules for each node, which is equivalent to a battery of 5.000 mAh with a 5 V output. Different energy levels (% of battery) were defined for each OBU at the beginning of the simulation to evaluate the implemented forwarding strategy that depends on a threshold that delimits the low battery, and the battery levels were placed in the nine Consumers (mobile nodes) of the Aveiro scenario in the following order: 90%, 10%, 55%, 14%, 32%, 88%, 23%, 45% and 76%. For 80 Consumers (mobile nodes) of the Porto scenario, we have used the following battery pattern (first 10 nodes and repeats the cycle for the remaining ones): 90%, 56%, 35%, 55%, 19%, 32%, 88%, 20%, 45% and 76%.

In order to evaluate the proposed forwarding strategy, four key metrics were considered [33]: satisfied Interest rate, transmission delay and network overhead (through In/Out Interests and In/Out Data). In order to evaluate the energy saving of the network, we considered the total energy consumed in each PHY state. In order to have a better reference regarding the performance of our solution, called Efficient version, we compared our results with the results obtained by another NDN implementation (Integrated version [32]) that has similar characteristics but without any energy management.

The configuration of each network element is described in Table 2. It was assumed that mobile nodes have a limited cache size; thus, many cache replacements will occur throughout the simulation. As the OBUs are network elements that have limited resources, due to their mobility capability, they will obviously have a limited battery capacity and with that also a lower caching capacity compared to the static network elements. Finally, OBUs were separated into Consumers and intermediate nodes.

The Consumer nodes will request all the available content related to each domain, both Urgent (U) and Non Urgent (NU), with a frequency of one Interest per second, to ensure that the requests are being constant and equal for all metrics. The content requests popularity follows a Zipf–Mandelbrot Distribution [34]. The Producer nodes will provide 5 chunks for each content in the domains of Emergency, Decision, Feedback and Interaction, and 10 chunks for each content in the domain of Information, which is the content domain that comprises most of the communications within the Internet. All these contents have freshness values increasing in relation to the domains. About 900 s for content within the Emergency domain, 1800 s for Decision content, 3000 for Feedback and Interaction contents and 3600 for Information contents. Contents that have a higher update rate and that are volatile have their freshness reduced so that they are not kept as long in Content Stores. For example, longer freshness for contents within the Information domain (about 3600 s, equivalent to one hour which is a quarter of the simulation time), because this type of content is not so volatile and therefore it can stay longer in the Content Stores.

### 5.1. Satisfied Interests Rate

**Figure 8:**
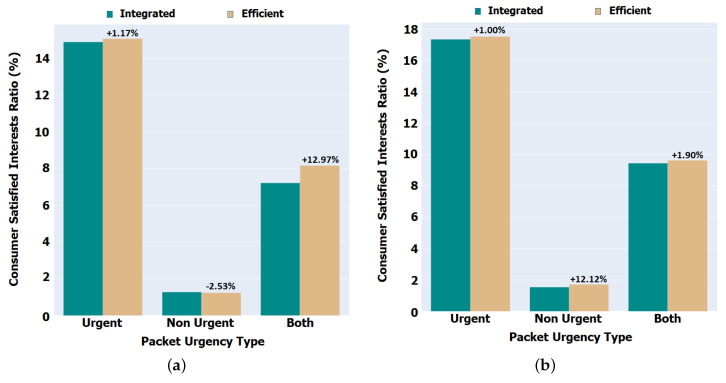
Satisfied Interest Rate (Urgent and Non Urgent). (**a**) Aveiro scenario. (**b**) Porto scenario.

In the Aveiro scenario, illustrated in Figure 8a, it can be observed that our implementation brought an increase of about 13% in the satisfaction of Interests in comparison with the Integrated version, either in urgent and non urgent packets. The difference comes from the fact that our implementation does not forward non urgent packets in some specific situations; hence, there is an increase for urgent packets and a decrease in the non-urgent packets. As a node goes into sleep mode whenever the surrounding environment justifies it, it is expected that there is a lower number of expired Interests, due to reverse path partitioning or the broadcast storm problem that happens in this highly mobile environment where Data follows the reverse path.

In the Porto scenario, illustrated in Figure 8b, our proposal had an increase of about 2% in the satisfaction ratio of Interests in comparison with the Integrated version, either in urgent and non urgent packets. The difference in the number of satisfied Interests between this use case and the Aveiro’s scenario (about 13%) is explained by the fact that, in the Porto scenario, as there is composed by buses moving at high speed compared to the boats, there is a higher mobility and consequently more intermittent disconnections between the nodes. That is, the contact between the nodes is faster and in a shorter time, while the slower boats have a longer connection between the nodes.

### 5.2. Transmission Delay

**Figure 9:**
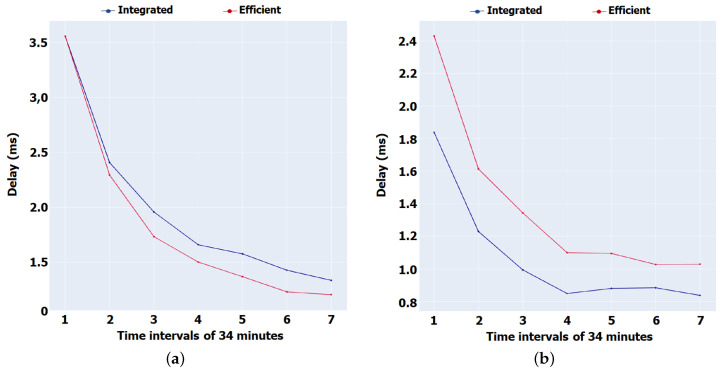
Transmission delay. (**a**) Aveiro scenario. (**b**) Porto scenario.

Figure 9 depicts the delay between the transmission of an Interest and the reception of the respective Data (boat scenario), during a period of 14,400 s divided into 34 min (2057 s) periods.

Our proposal shows, for most of the time, an increase in performance, showing slightly lower performance only in the first 85 min (2.5 interval) in relation to the Integrated version. The results obtained reveal that the content is closer to the Consumers since a Leave-Copy-Everywhere approach is followed in the cache placement. To complement this, the number of Interests circulating through the network is smaller, which justifies the fact that the content is closer to the Consumers. The fact that the nodes enter in Sleep mode when they are in adverse conditions contributes to this, thus reducing the number of packets transmissions that most likely would be expired and lost in the network.

On the other hand, the delay observed in the Porto scenario (Figure 9b) presents a higher delay in our version because as the nodes are in a high mobility environment, most of them will be constantly faced with adverse situations that imply immediate falling asleep for a short period of time (1 s). During this period, a node that possibly in the Integrated version would be awake and could be part of the return route of data to a particular Consumer, would no longer receive packets, interrupting the of data, or Interests to their destinations. This factor leads that the full delay and last delay present higher values with our solution.

### 5.3. Network Overhead

**Figure 10:**
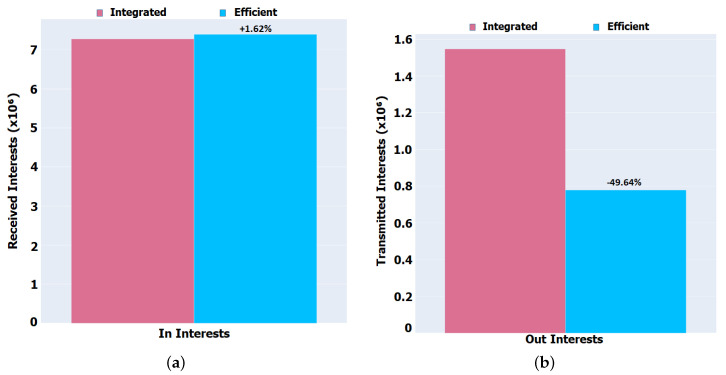
Global received and transmitted Interests packets for Porto scenario. (**a**) In Interests. (**b**) Out Interests.

Through Figure 10, we can evaluate the network overhead for the Porto scenario. It is possible to see that the overall number of Interests flowing through the network has increased by about 2% for In Interests (Integrated = 7,277,690, Efficient = 7,395,351) and reduced by about 50% for Out Interests (Integrated = 1,547,405, Efficient = 779,320) with the new implementation. The increase in the Interests received is due to the fact that there is more mobility in this scenario, being the contact between the nodes for the exchange of information reduced, so there will be packets that will be lost in the network or that will expire, originating retransmissions to obtain the content. On the other hand, the transmitted Interests are reduced by the reduction in the time in Idle Mode with the sleeping of the nodes and the fact that there are more nodes in the neighbourhood. Since sending only one packet through broadcast for the entire neighbourhood, all nodes will receive the same packet increasing the In Interest number, so there is an increase in In Interest and a reduction in Out Interests.

**Figure 11:**
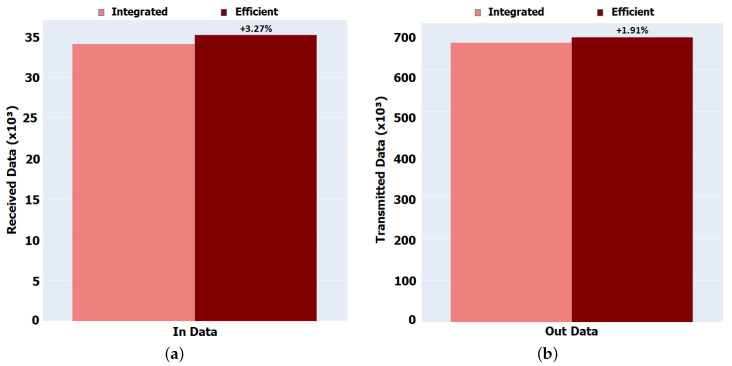
Global received and transmitted Data packets for Porto scenario. (**a**) In Data. (**b**) Out Data.

Regarding the Data packets, illustrated in Figure 11, it can be observed that although there are fewer Interest packets being transmitted due to the short contact and intermittent disconnections between nodes, there are more Data packets moving around the network. This attests that the Interests that were transmitted were successfully satisfied. In the Integrated version, the number of Interests satisfied is less, meaning that they could expire even before receiving Data, which resulted in discarding Data packets and consequently reducing their transmission by other nodes. Our version shows an increase in Data packets circulating in the network by 3.3% for In Data (Integrated = 34,073, Efficient = 35,188) and by 2% for Out Data (Integrated = 664,074, Efficient = 676,766).

In the Aveiro scenario, the total number of Interests flowing through the network has been reduced by about 0.02% for internal Interests and about 38% for external Interests with our implementation. This is mainly due to the fact that a node reduces its time in idle mode and goes to sleep when its internal state and the environment demands it, producing and receiving fewer Interest packets than in the Integrated version. In the Integrated version, the number of Interests satisfied is less, meaning that they could expire even before receiving Data, which resulted in discarding Data packets and consequently reducing their transmission by other nodes. The Efficient version shows an increase in Data packets circulating in the network by 0.90% for both In Data and Out Data.

Despite having a greater delay in response to the Data, the few Interests that are sent are satisfied by Data, attesting that our solution has better performance in terms of not forwarding unnecessary packets and reducing idle times, which will be reflected in the times in each state and energy saving.

### 5.4. Energy Consumption

**Figure 12:**
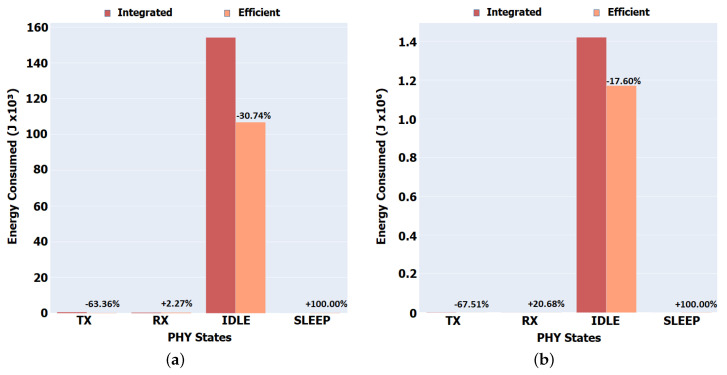
Total energy consumed in each PHY state. (**a**) Aveiro scenario. (**b**) Porto scenario.

The results presented in Figure 12a show a reduction of around 63% (Aveiro scenario) of the energy and time spent in the transmission of packets (TX Mode) in the Efficient version (Integrated TX = 587, Efficient TX = 215). Considering the increase in energy and time spent in the reception of packets (RX Mode), it can be concluded that the large reduction in energy and time spent in both idle Mode and TX Mode impacted only the packets that would be lost in the network because they could never reach the nodes, ensuring an almost accurate transmission and reception of the packets that matter and that actually have a chance of reaching their destination.

The increase in the time in receiving mode (Rx) is due to the fact that there are fewer packets circulating in the network, meaning that the increase in the reception energy consumed is for the Data packets that successfully reach the Consumer. A reduction of between 17% (Porto scenario—Integrated IDLE = 1,420,309, Efficient IDLE = 1,170,393) and 30% (Aveiro scenario—Integrated IDLE = 154,149, Efficient IDLE = 106,768) in the energy consumption performed in idle mode is also visible, as the time spent in this state was converted into consumption in Sleep Mode.

With the Efficient implementation, a node goes to sleep mode depending on its internal energy state (battery level) and the environment it is in. This reflects in a reduction in the number of transmissions and receptions. However, a node wakes up whenever it produces an Interest request to be sent to the network, waking up the node for that purpose. The reduction in transmission energy between the Integrated and Efficient version is directly related to the reduction in the number of Interests circulating in the network, because the content is closer to the Consumers.

Regarding the network lifespan, which is due to the energy savings considered by the forwarding strategy and the sleep and wake mechanisms, there is more energy distributed to the mobile nodes of the network in our Efficient version. Our results show a 1.3% increase for the Aveiro scenario (boats) and a 1.5% increase for the Porto scenario (buses). These savings translate into an additional 25 min of network activity for the Aveiro scenario and 4 h and 40 min for the Porto scenario.

## 6. Conclusions

In this paper, we are proposing a forwarding strategy for energy-constrained wireless ICN networks. Our solution considers the mobility of the nodes, which directly impacts the forwarding decision of a packet and checks the environment around the node, which includes the number and type of neighbours, signal strength and direction of movement. Furthermore, it combines this information with the internal state of each node, both in its internal energy level and in state transitions between awake and sleep. The proposed forwarding strategy extends and adapts concepts of ICNs, by means of packet domain analysis, neighbourhood evaluation and node sleeping and waking strategies so that there is energy saving and non-use of resources in unnecessary situations, such as the moments when a node is listening when it is isolated. In addition, our proposal guarantees the privacy and security of information that ends up not being shared, such as their neighbourhood and their energetic state. We also ensure that packets are prioritised with a major impact on how the routing decision and sleeping strategy is made.

The evaluation of our solution was performed by simulation with real traces of urban mobility in two distinct scenarios: boats in the lagoon of Aveiro and public buses in the city of Porto, both in Portugal. These scenarios offer different conditions. Boats sailing in low speed represent less mobility and a longer contact time between the several nodes, while buses circulate at a higher speed, leading to a shorter time of contact between the several nodes due to intermittent and faster disconnections. Our tests considered the dissemination of Urgent and Non Urgent packets to evaluate the satisfied Interest rate, transmission delay, network overhead and energy consumption, important metrics used in ICN/NDN performance network evaluation.

Our results have shown that the proposed forwarding strategy resulted in significant improvements in network content availability, overall node power savings and increased network lifetime. In addition, we have shown that it was possible to save energy that was wasted listening and forwarding packets unnecessarily, generating a longer lifetime distributed by the network nodes, which was more than 25 min in the case of Aveiro and it was almost 5 h of network activity in the case of Porto.

As future work, we intend to expand our solution by adding mobile node speed assessment, handling for multiple communication interfaces and a dynamic low battery threshold among other improvements.

## Figures and Tables

**Table 1:** Parameters used in the Aveiro and Porto mobile scenarios.

Parameter	Aveiro	Porto
**Number of nodes**	15	100
**OBUs**	9	80
**RSUs**	4	26
**Backend Routers (BR)**	2	4
**Content size**	1024 bytes	1024 bytes
**Cache size**	20 OBUs, 60 RSUs, 100 BR	20 OBUs, 60 RSUs, 100 BR
**Cache eviction policy**	LRU	LRU
**Forwarding strategy**	Best-Route Modified	Best-Route Modified
**Propagation delay model**	ConstantSpeedPropagation	ConstantSpeedPropagation
**Propagation loss model**	RangePropagationLoss	RangePropagationLoss
**Simulation time**	14,400 s	14,400 s

**Table 2:** Network element’s configuration.

	OBUs		RSUs	Routers
**Node Type**	Consumer	Intermediate	Producer	Backend
**Installed** **Technologies**	Wi-Fi 802.11n		Wi-Fi 802.11n Ethernet	Ethernet
**Mobility Type**	Mobile		Static	
**Cache Size**	20	20	60	100
**Aveiro’s Devices**	9	0	4	2
**Porto’s Devices**	40	40	26	4
